# Viral metagenomic analysis of chickens with runting-stunting syndrome in the Republic of Korea

**DOI:** 10.1186/s12985-020-01307-z

**Published:** 2020-04-15

**Authors:** Hye-Ryoung Kim, Yong-Kuk Kwon, Il Jang, You-Chan Bae

**Affiliations:** Animal and Plant Quarantine Agency, 177 Hyeoksin 8-ro, Gimcheon-si, Gyeongsangbuk-do 39660 Republic of Korea

**Keywords:** Viral enteritis, Runting-stunting syndrome, Metagenomics, RNA virus

## Abstract

**Background and aims:**

Runting-stunting syndrome (RSS) in chickens, also known as malabsorption syndrome, which is characterized by mild to severe enteritis and diagnosed through typical histopathologic examination as well as clinical signs, results in considerable economic losses. Despite the many studies carried out over decades to determine the etiologic agents of RSS involved in the disease, several outbreaks remained without the elucidation of, potentially multiple, etiologies involved.

**Methods:**

We performed comparative analysis of viral metagenomes from four chicken flocks affected with RSS using next-generation sequencing. Primers for the detection of chicken enteric viruses were designed from the sequencing data obtained with metagenomics. Multiplex reverse transcription–polymerase chain reaction (PCR) and PCR were performed to detect a variety of etiological agents previously described in natural cases of RSS.

**Results:**

The most abundant viral families identified in this study were *Astroviridae*, *Picornaviridae*, *Parvoviridae*, *Caliciviridae*, *Reoviridae* and *Picobirnaviridae*. Chicken astrovirus sequences were present in all four samples, suggesting an association between chicken astrovirus and RSS and chicken astrovirus as a candidate pathogen responsible for RSS. Picobirnavirus and the newly identified chapparvovirus were found in chickens in the Republic of Korea for the first time, and the genetic diversity of enteric viruses and viral communities was showed.

**Conclusions:**

Chicken astrovirus was consistently detected in broilers affected with RSS and the result of this study may contribute to knowledge of enteric diseases and viruses in chickens.

## Introduction

Infectious intestinal diseases affecting young chickens and turkeys are characterized by mild to severe enteritis and result in considerable economic losses. Viral enteritis increases susceptibility to other diseases, decreases feed conversion efficiency, and prolongs the time to market [1, 2]. Runting-stunting syndrome (RSS), also known as malabsorption syndrome, is diagnosed based on clinical signs (diarrhea, dehydration, growth depression, unevenness in size) and histopathological lesions (distension of the crypts of the small intestine, inflammatory cell infiltration in the lamina propria adjacent to affected crypts and necrotic cellular debris in crypts) [3, 4].

Over the years, many studies have attempted to elucidate the etiology of RSSs and reovirus, rotavirus, parvovirus, and astrovirus have been suggested as the most likely etiologic factor. Infectious bronchitis virus and fowl adenovirus have also been implicated [5–9]. It is usually difficult to isolate these enteric viruses in vitro, and many studies with astrovirus, rotavirus and reovirus have reproduced intestinal disease in chickens [8–16]; however, the exact etiological agents of RSS remain controversial.

Metagenomics provides the opportunity to simultaneously detect a large number of sequences from different microbes, including viruses [17]. Metagenomics has enabled a recent increase in understanding of viral diversity, and environmental and clinical metagenomics have contributed to the discovery of novel viruses [18]. In addition, studies are necessary to determine sequence differences by comparison with high-throughput data obtained from a variety of genomic regions, and samples through various protocols that differ in terms of sample preparation, cDNA synthesis, library preparation, sequencing and data analysis [19].

Here, Illumina sequencing was applied to explore viral communities in the small intestines and intestinal contents of four chicken flocks with RSS and one specific pathogen-free (SPF) chicken flock in the Republic of Korea.

## Methods

### Samples and purification

The small intestines and intestinal contents of broiler chickens from four flocks diagnosed with RSS (05D72, 07D11, 13D62 and 13Q45) were inspected in this study. The chickens were submitted to the Avian Disease Division (ADD) of the Animal and Plant Quarantine Agency (APQA) for disease diagnosis between 2005 and 2013 and diagnosed by an APQA diagnostic protocol on the basis of clinical manifestation and the presence of gross lesions. The small intestines (duodenum, jejunum and ileum) of broiler chickens in flocks 05D72 and 07D11 were collected after necropsy, processed promptly via blending into a 10% homogenate in sterile phosphate-buffered saline (PBS) containing 0.4 mg gentamicin per ml, and stored at − 80 °C until analysis. The intestinal contents of broiler chickens in flocks 13D62 and 13Q45 and control (SPF) chickens were collected from the small intestine after necropsy, mixed with an equal volume of sterile PBS containing 0.4 mg gentamicin per ml, and stored at 2~5 °C until analysis. RSS-positive and control samples were centrifuged at 3500 r.p.m. and 13,000 r.p.m. for 10 min each. To remove large particles and bacteria, the supernatants of intestine homogenates were filtered with 0.8, 0.45, and 0.22 μm syringe filters, and feces filtrates were concentrated using an Amicon ultrafiltration apparatus (Amicon chamber 8400 with a 30 kDa MWCO UF membrane, Millipore, USA).

Viral particles were pelleted by ultracentrifugation (30,000 r.p.m., 5 h, 4 °C), resuspended in 500 μL of 1 M Tris-Cl (pH 7.4) and treated with 2.5 units of DNase I (AMPD1, Sigma-Aldrich, USA) for 3 h at 37 °C to eliminate free DNA. The samples were concentrated and washed twice using a Microcon 30 column (Millipore, USA). DNase activity was inhibited by adding 0.5 M EDTA to a concentration of 20 mM.

### High-throughput sequencing and analysis

Total RNA was extracted from the purified samples using a Viral Gene-spin viral DNA/RNA extraction kit (iNtRON Biotechnology, Republic of Korea) according to the manufacturer’s instructions. cDNA synthesis and PCR amplification of nucleic acid were carried out in a 50 μL mixture containing 5 μg of RNA, 0.5 μM random primer K (GAC CAT CTA GCG ACC TCC AC) and 0.5 μM primer KN (GAC CAT CTA GCG ACC TCC CAN NNN NNN N) as described previously [20] using the Access RT-PCR system (Promega, USA). The products were purified using an UltraClean PCR Clean-Up Kit (MO BIO, USA) and sequenced at Theragen Etex (Suwon, Republic of Korea). Sample libraries were prepared using an Illumina TruSeq DNA sample preparation kit (Illumina, USA), and DNA was fragmented using a Covaris adaptive focused acoustics device to generate double-stranded DNA fragments 300–400 bp in size. The ends were repaired, phosphorylated, and 3′-end adenylated. Paired-end DNA adaptors (Illumina) were ligated, and the resulting construct fragments ~ 500 bp size were selected. Libraries were loaded onto a paired-end flow cell and sequenced as 101 bp paired-end, indexed reads on an Illumina HiSeq 2000 instrument. The raw read sequences were filtered using the following criteria: 1) the presence of ambiguous bases (letter N) in excess of 10%, 2) an average quality below 20, 3) more than 5% of nucleotides with quality inferior to 20, and 4) the presence of an adapter sequence (Supplementary data 1). Host genome (galgal4)-filtered reads were aligned with known viral, bacterial, and fungal genome database from NCBI using Burrows-Wheeler Aligner software. Data from each sample were assembled using MetaVelvet (k-mer size 51) and Bambus2. Homology-based (BLAST) classification based on the nucleotide sequence was performed using the ‘nucleotide’ database from NCBI to annotate the scaffolds.

### Sequence analysis

Nucleotide and amino acid sequences were aligned using Vector NTI 10 software. The aligned fragments from *Astroviridae, Picornaviridae, Parvoviridae, Caliciviridae* and *Picobirnaviridae* were trimmed, based on lengths of 302, 1421, 3661, 7315, and 812 bp, respectively, using BioEdit software. Phylogenetic trees were generated by the neighbor-joining method using MEGA 4.0 software [21] with 1000 bootstrap replications. Assignment of genotypes to rotavirus A sequences was performed using the online tool RotaC [22].

### Trial for whole-genome analysis of a parvovirus

The design of primers for bridging contigs identified by Illumina, and the resequencing of these regions, were carried out at Cosmo Genetech Co., Ltd. (Seoul, Republic of Korea) using an ABI 3730 DNA sequencer. Nested PCR and rapid amplification of cDNA ends (RACE) [23] were attempted to obtain sequences of the 5′ and 3′ ends of a parvo-like virus genome.

### Detection of enteric viruses

Sequences revealed through high-throughput sequencing indicated a variety of etiologies related to RSS. Multiplex reverse transcription (RT)–polymerase chain reaction (PCR) and PCR were used to distinguish these agents from the original chicken intestine homogenate using primer sets. Most primers were designed using CLC Main Workbench 6 with the high-throughput sequences obtained in this study, and the primers for rotavirus D used were chosen according to a published report [24], as described in Table 1. DNA and RNA from individual enteric samples were extracted using a Viral Gene-spin Viral DNA/RNA Extraction kit (iNtRON Biotechnology, Republic of Korea) according to the manufacturer’s instructions. Multiplex RT-PCR for the detection of picornavirus, astrovirus and calicivirus was carried out using the PrimeScript One Step RT-PCR kit ver.2 (TaKaRa, Japan) following the manufacturer’s instructions, with each primer at 0.5 μM and 2 μL of RNA. Thermocycling conditions were as follows: 50 °C for 30 min and 94 °C for 2 min, followed by 40 cycles of 94 °C for 30 s, 50 °C for 30 s, and 72 °C for 1 min, and a final step at 72 °C for 5 min. Multiplex RT-PCR for the detection of rotaviruses A, D and F was carried out using the PrimeScript One Step RT-PCR kit ver.2 (TaKaRa), with each primer at 0.5 μM and 2 μL of RNA. Thermocycling conditions were as follows: 50 °C for 30 min and 94 °C for 2 min, followed by 40 cycles of 94 °C for 30 s, 60 °C for 30 s, and 72 °C for 1 min and a final step at 72 °C for 5 min. To detect the parvovirus, PCR was performed using AccuPower PCR Premix (Bioneer, Republic of Korea) with the following conditions: 95 °C for 5 min; 30 cycles of 94 °C for 30 s, 55 °C for 1 min, and 72 °C for 1 min; and final incubation at 72 °C for 5 min. The sensitivity and specificity of the primer pairs for each agent were tested by uniplex PCR. All PCR products were purified from agarose gels using a QIAquick gel extraction kit (Qiagen, USA) and sequenced directly using an ABI 3730 DNA sequencer at Cosmo Genetech Co., Ltd. using the respective PCR primers.

**Table 1:** Primers and PCR instrumental conditions for detecting enteric viruses

Virus	Target gene	Primer sequences	PCR condition	Product size (bp)
parvovirus	NS	PV-CLC-F1 5′-ACGAAGGTAAGAATGAGG-3′ PV-CLC-R1 5′-GGAGATTGATTGGGGATG-3’	PCR 95 °C, 5 min - 30 cycles(94 °C, 30s, 55 °C, 1 min, 72 °C, 1 min) - 72 °C, 5 min	364
picornavirus	NS	Picorna-F3 5’-TACCCGAGAAAACGACCC-3′ Picorna-R3 5′-ACACCTCAGCTACAAGAA-3’	Multiplex RT-PCR 50 °C, 30 min - 94 °C, 2 min - 40 cycles(94 °C, 30s, 50 °C, 30s, 72 °C, 1 min) - 72 °C, 5 min	504
astrovirus	ORF1a	Astro-F1 5’-ATTCCAGTTCAGCTCTTC-3′ Astro-R1 5′-TCCTGTTGGTAAGAGAGT-3’		138
calicivirus	VP1	Calici-CLC-F3 5’-AGAAGCGTGACTACATGGA-3′ Calici-CLC-R1 5′-TTGTGTTGTTTGTGGGGT-3’		1247
rotavirus A	VP6	RotaA-CLC-F2 5’-CTCCTCAATCTAATGCACT-3′ RotaA-CLC-R2 5′-CCGAACCATTATTTAGCCA-3’	Multiplex RT-PCR 50 °C, 30 min - 94 °C, 2 min - 40 cycles(94 °C, 30s, 60 °C, 30s, 72 °C, 1 min) - 72 °C, 5 min	230
rotavirus D^a^	VP6	RotaD-F 5’-GGAGGCGCTGTCTTCAATTGCG-3′ RotaD-R 5′-TGGCCAATAGTGTGTGGCAGCT-3’		742
rotavirus F	VP6	RotaF-CLC-F2 5’-GTGGAGTCGGAAATATGG-3′ RotaF-CLC-R2 5′-CAGGTACAACATAAGGATTC-3’		330

^a^primers for detecting rotavirus D were used according to the reference [14]

These detection methods were used to identify the distribution of these enteric viruses in non-RSS-infected chickens. Intestinal samples were randomly collected from 86 non-RSS-infected broiler chickens less than 6 weeks old submitted to the ADD of the APQA for diagnosis. These specimens were diagnosed with a variety of diseases, viral diseases (infectious bronchitis, inclusion body hepatitis, infectious bursal disease, etc.), bacterial diseases (necrotic enteritis, bacterial arthritis, colibacillosis, etc.), or complicated disease. Multiplex RT-PCR and PCR were carried out as described above.

## Results

### Diagnosis of RSS

**Fig. 1:**
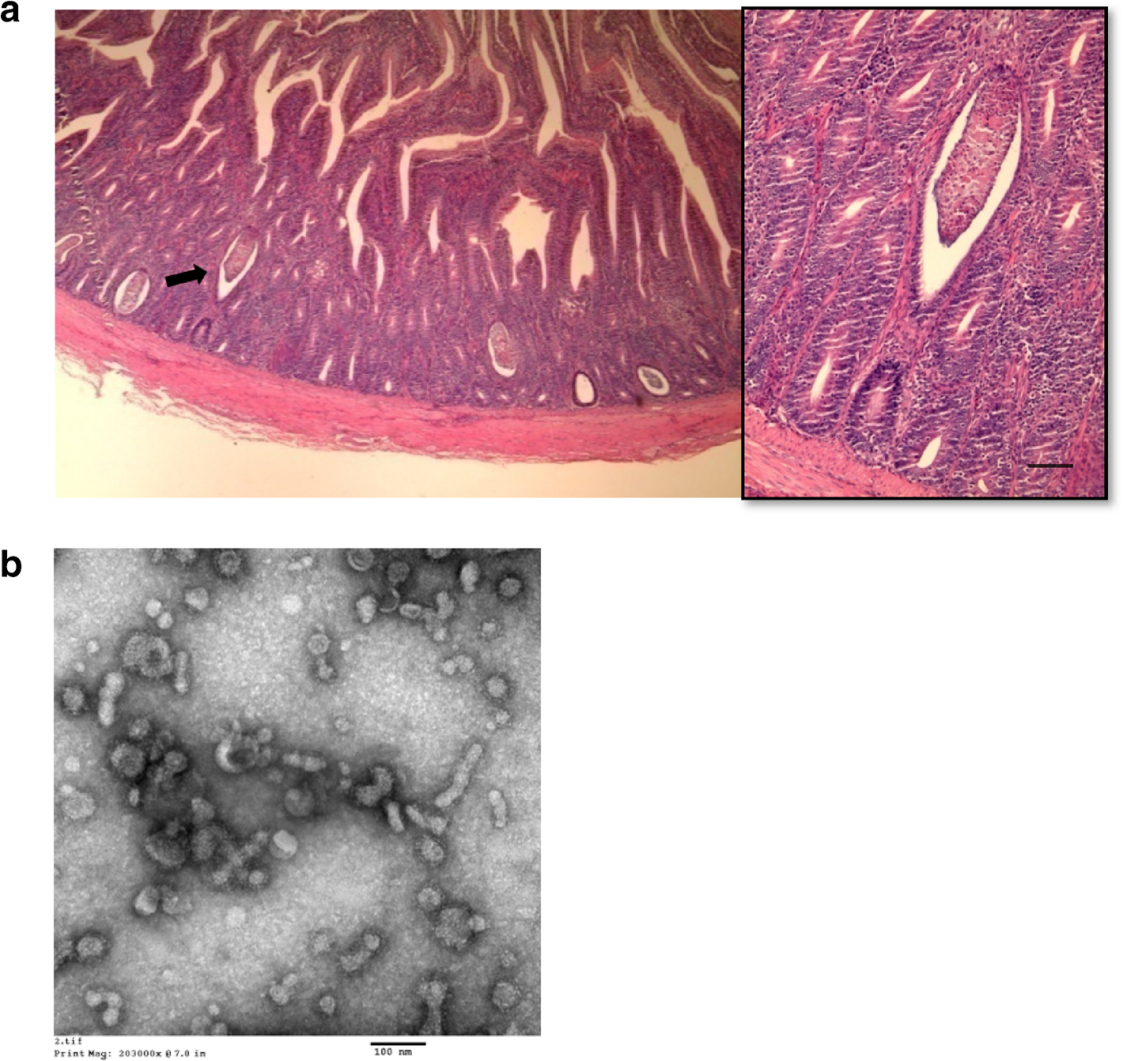
Diagnosis of runting-stunting syndrome using (**a**) histopathological lesions showing distension of the crypts in the small intestine lined with a flattened epithelium, exfoliated cells in the crypts and inflammatory cell infiltration in the adjacent lamina propria (100×). The black arrow indicates that the crypt contains necrotic cellular debris observed at higher magnification (right, 400×). Bar indicates 25 μm. **b** Negative contrast electron micrograph of feces and intestine homogenates of chickens with viral enteritis

The four flocks tested comprised 2-week-, 3-week-, 4-week-, and 6-week-old broiler chickens with poor growth, retarded feathering, diarrhea and various clinical signs. Thin-welled intestines filled with undigested feed at postmortem examination and characteristic microscopic lesions (distension of the crypts in the duodenum and jejunum lined with flattened epithelium, exfoliated cells in the crypts and inflammatory cell infiltration in the adjacent lamina propria) were observed, but villous atrophy was not observed (Fig. 1a). One flock (07D11) was diagnosed with only RSS, whereas the other three flocks (05D72, 13D62 and 13Q45) had been infected with additional diseases, such as infectious bronchitis (IB), inclusion body hepatitis (IBH) and coccidiosis (Table 2). IB was identified by virus isolation and RT-PCR, IBH was confirmed by PCR and histopathology (observation of intranuclear inclusions in the liver), and coccidiosis was confirmed by the presence of oocysts in the cecum using microscopic examination. Electron microscopy identified many small, round and nonenveloped viral particles of various sizes in all four samples (Fig. 1b).

**Table 2:** Summary of chicken samples used in the study

ID	05D72	07D11	13D62	13Q45	Control
**year**	2005	2007	2013	2013	–
**species**	Broiler	Broiler	Broiler	Broiler	SPF
**age (weeks)**	6	2	3	4	4
**final diagnosis**	RSS, IB	RSS	RSS, IBH	RSS, Coccidiosis	–
**morbidity (%)**	uk	uk	48.6	52.5	–
**sample**	small intestine	small intestine	intestinal contents	intestinal contents	intestinal contents
**total reads**	14,893,900	17,152,624	19,711,846	18,325,438	18,906,096
**No. of sequences**	18,777	57,429	42,806	20,526	60,556
**mean length**	182	192	176	162	193
**total viral reads**	226,870	162,917	1,766,642	48,979	339,024
bacteriophage	7463 (3.3)^a^	2618 (1.6)	109,672 (6.2)	10,337 (21.1)	339,024 (100)
*Leviviridae*	7463	1937	17,237	3836	270,026
*Siphoviridae*	–	–	82,207	3248	71
*Myoviridae*	–	–	6035	36	3731
*Podoviridae*	–	–	907	3118	5433
unassigned phage	–	684	3286	99	3898
*Astroviridae*	128 (0.1)	3424 (2.1)	1,328,548 (75.2)	4208 (8.6)	–
*Picornaviridae*	214,648 (94.6)	39,646 (24.3)	207,500 (11.8)	18,567 (37.9)	–
*Parvoviridae*	–	9563 (5.9)	13,793 (0.8)	2599 (5.3)	–
*Reoviridae*	–	107,666 (66.1)	67,458 (3.8)	–	–
*Caliciviridae*	–	–	19,802 (1.1)	7822 (16.0)	–
*Picobirnaviridae*	–	–	16,128 (0.9)	5446 (11.1)	–
*Adenoviridae*	–	–	3741 (0.2)	–	–
*Coronaviridae*	409 (0.2)	–	–	–	–
*Anelloviridae*	4222 (1.9)	–	–	–	–

uk: unknown

*RSS* Runting-stunting syndrome, *IB* Infectious bronchitis, *IBH* Inclusion body hepatitis

^a^The percentage of detected virus reads/total virus reads were indicated in the parenthesis

### Metagenomics data analysis

Five samples (05D72, 07D11, 13D62, 13Q45 and control) were sequenced, producing a total of 14,893,900–19,711,846 reads with an average read length of 162–193 bp, representing a total of 18,777–60,556 sequences. Homology-based (BLAST) classification showed a total of 48,979-1,766,642 viral sequence reads and identified ten different viruses with sequence identity to avian viruses and bacteriophages. Bacteriophage sequences comprised 3.3 to 100% of the viral sequences and were assigned to known families, including *Leviviridae, Siphoviridae, Myoviridae* and *Podoviridae*. Most of the samples had abundant *Leviviridae* sequences, but 13D62 and 13Q45 showed plentiful *Siphoviridae* sequences. All samples excluding 05D72 contained sequences from unassigned phages. The identified viral sequences were assigned to known viral families, including *Picornaviridae, Astroviridae, Parvoviridae, Reoviridae, Caliciviridae, Picobirnaviridae, Adenoviridae, Coronaviridae* and *Anelloviridae*. In all four samples from chickens diagnosed with RSS, *Astroviridae* and *Picornaviridae* were detected. *Parvovirida*e sequences were detected in three (07D11, 13D62, 13Q45) of the four samples, *Reoviridae* sequences were identified in two samples (07D11, 13D62)*,* and *Caliciviridae* and *Picobirnaviridae* sequences were detected in two samples (13D62, 13Q45). *Coronaviridae* and *Anellovir*i*dae* sequences were identified in sample 05D72, and *Adenoviridae* sequences were identified in sample 13D62. Sample 05D72 from chickens diagnosed with infectious bronchitis as well as RSS contained *Coronaviridae* (0.2%) with *Picornaviridae* (94.6%), *Astroviridae* (0.1%) and *Anelloviridae* (1.9%). *Reoviridae* (66.1%) and *Picornaviridae* (24.3%) families were abundant in sample 07D11, which also contained *Parvoviridae* (5.9%) and *Astroviridae* (2.1%) sequences. Sample 13D62, which was taken from chickens with a final diagnosis of inclusion body hepatitis and RSS, contained *Astroviridae* (75.2%) and *Picornaviridae* (11.8%) sequences, followed by sequences from *Reoviridae* (3.8%), *Caliciviridae* (1.1%), *Picobirnaviridae* (0.9%), *Parvoviridae* (0.8%) and *Adenoviridae* (0.2%) families at equal abundance. Sample 13Q45 showed a viral profile similar to that of sample 07D11 containing *Picornaviridae* (37.9%), *Astroviridae* (8.6%), *Parvoviridae* (5.3%), *Caliciviridae* (16.0%) and *Picobirnaviridae* (11.1%) sequences, but no sequences were assigned to *Reoviridae.* Sequences from the control sample differed from those of all other samples and contained no viral reads assigned to known avian viruses and only bacteriophage sequences (Table 2 Supplementary data 2).

### Enteric virus genome sequence analysis

Sixty-six contigs from six viral families 251 to 7315 nucleotides (nts) in length were obtained from the four RSS-positive chicken samples. These genome sequences were deposited into GenBank under accession numbers KM254161-KM254224.

#### Astroviridae

**Fig. 2:**
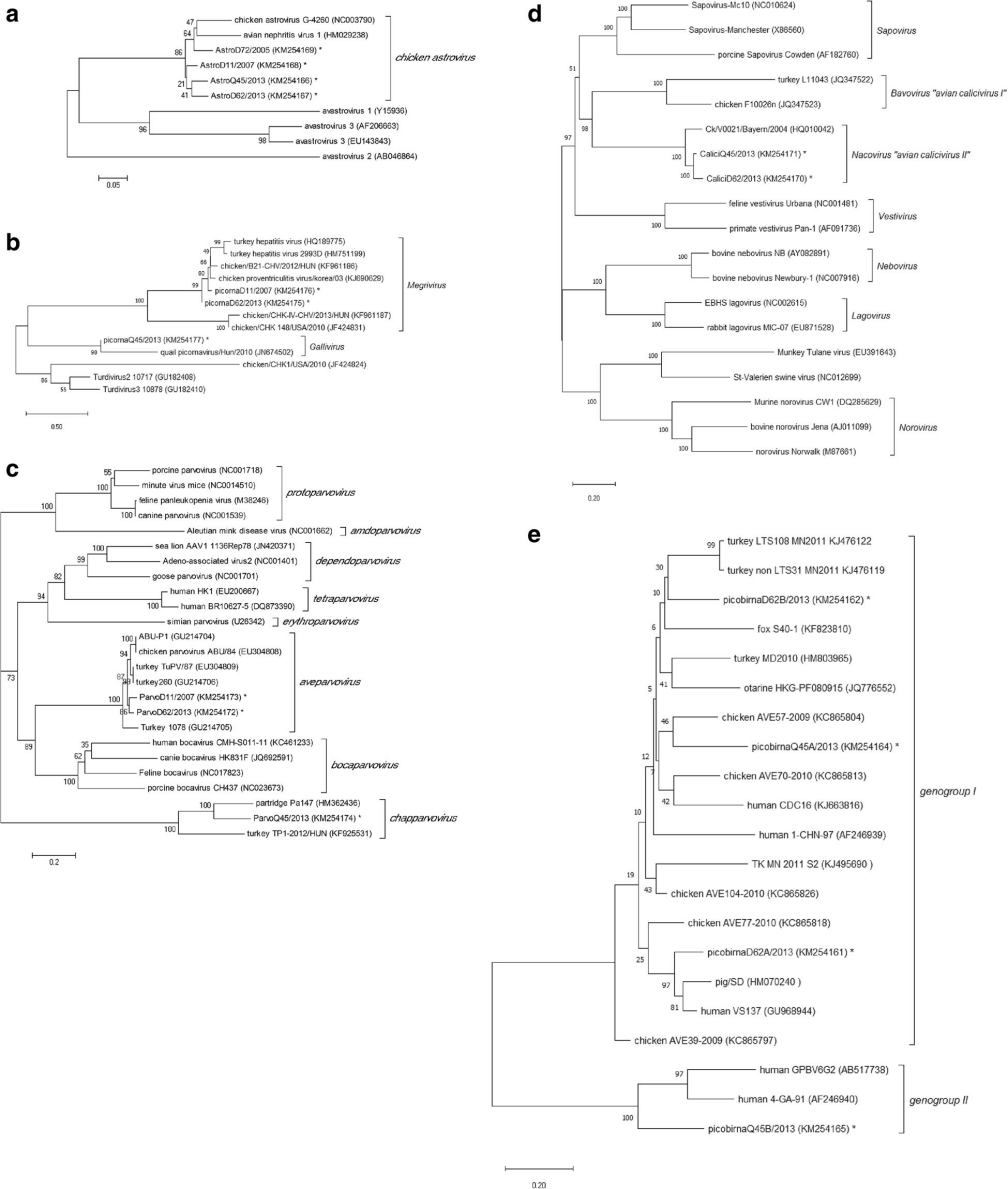
Phylogenetic analysis of viruses among (**a**) *Astroviridae*, (**b**) *Picornaviridae*, (**c**) *Parvoviridae*, (**d**) *Caliciviridae*, and (**e**) *Picobirnaviridae* identified from chickens with runt-stunting syndrome. A phylogenetic tree was generated using the neighbor-joining method and used to compare individual sequence reads to corresponding sequences from known viruses. Bootstrap values were deduced from 1000 replicates. The viruses that were identified in this study are highlighted with asterisk

Four contigs with similarity to genomes of the Avastrovirus genus were identified; specifically, chicken astrovirus sequences were found in all four samples. The contigs identified in samples 05D72, 07D11 and 13D62 were partial non- structural (NS) polyprotein 302, 305, and 2266 nt in length, respectively. The 13Q45 contig consisted of full NS and partial capsid protein sequences 5084 nt in length. The four NS sequences from the chicken astrovirus genome detected showed 84.2~86.7% nt homology to the Chinese strain (GenBank No. HM029238) [25] and were clustered differently from other Avastrovirus genus (avastrovirus 1, 2, and 3) in the phylogenetic tree (Fig. 2a).

#### Picornaviridae

Four *Picornaviridae* contigs were recovered from RSS-positive samples. The 07D11 and 13D62 contigs showed higher similarity to chicken megrivirus (GenBank No. KJ690629), but the 05D72 and 13Q45 contigs were similar to chicken gallivirus (Fig. 2b).

#### Parvoviridae

Two nearly complete Aveparvovirus genome sequences 4782 and 5034 nt in length were identified from samples 07D11 and 13D62, respectively. Both contigs showed 92.7 and 97.1% nt identity with the ABU-P1 strain (GenBank No. GU214704) and contained three ORFs that encode nonstructural (NS1, NP1) and capsid (VP1, VP2) proteins. A parvo-like virus (3661 nt sequence length) was obtained from sample 13Q45 and found to contain the complete NS1 CDS (Fig. 2c). Sequence comparison with the ABU-P1 strain revealed a high level of divergence with 46.6% nt identify and 13.6% aa identify. The DNA coding sequences was confirmed by Sanger sequencing of amplicons obtained by primer walking. Nested PCR and RACE were conducted to obtain the sequences of the 5′ and 3′ ends of a parvo-like virus genome in sample 13Q45 but yielded no products. In the *Parvoviridae* phylogenetic tree, this identified virus was clustered with the recently classified genus Chapparvovirus [26].

#### Reoviridae

A total of 47 viral contigs belonging to three rotavirus species (rotaviruses A, D, and F) were detected in samples 07D11 and 13D62. All eleven genome segments of rotaviruses A, D and F were compared with those of the German strains 02V0002G3, 05 V0049 and 03 V0568. The sequences of contigs from rotavirus A obtained from sample 13D62 were similar to sequences of strain 02V0002G3 (GenBank No. FJ169853–169863), and each viral segment showed 91.2~96.3% nt identity with the corresponding segment in strain 02V0002G3, but the VP4 gene showed a low degree of identity (74.9%) and a different genotype (P31). The percent identities of rotavirus D segment-associated contigs with strain 05 V0049 (GenBank No.GU733443–733453) varied (79.5–95.5%). Rotavirus F segments from sample 13D62 showed between 72.6 and 91.7% nt identity to strain 03 V0568 (GenBank No. FJ169853–169863). The sequences of contigs from rotavirus species in sample 07D11 were similar to those of contigs in sample 13D62 (Table 3).

**Table 3:** Genome segment sizes and nucleotide sequence identities rotaviruses A, D and F derived from chicken with viral enteritis

Protein	Rotavirus A				Rotavirus D			Rotavirus F		
	Size in nucleotides (% nt identity^a^)			Geno-type	Size in nucleotides (% nt identity)			Size in nucleotides (% nt identity)		
	02V0002G3	13D62	07D11		05 V0049	13D62	07D11	03 V0568	13D62	07D11
VP1	3305	3253 (91.6)	–	R6	3274	3154 (90.2)	430 (90.2)	3296	3247 (89.6)	305 (87.6)
VP2	2732	2676 (94.1)	829 (94.8)	C6	2801	2460 (90.0)	806 (91.7)	2769	2382 (89.8)	–
VP3	2583	2517 (91.7)	–	M7	2366	2279 (81.5)	1520 (82.9)	2246	2075 (72.6)	–
VP4	2354	2251 (74.9)	–	P31	2104	1990 (95.5)	1094 (88.3)	2174	1848 (91.7)	–
NSP1	2122	1693 (91.2)	266 (92.2)	A16	1872	1676 (95.5)	590 (96.9)	1791	1657 (87.8)	–
VP6	1348	434 (96.3)	–	I11	1353	1265 (96.4)	343 (96.2)	1314	1250 (91.5)	–
NSP3	1089	1049 (90.2)	–	T8	1242	1110 (88.1)	570 (87.8)	1309	1197 (81.8)	–
NSP2	1042	675 (94.8)	–	N6	1026	773 (87.7)	567 (90.5)	1068	598 (90.7)	–
VP7	1066	1033 (94.4)	–	G19	1025	995 (86.6)	326 (88.7)	990	876 (76.5)	–
NSP4	724	251 (93.6)	–	E10	765	466 (93.1)	442 (92.8)	706	581 (86.1)	433 (89.4)
NSP5	699	272 (94.1)	–	H8	672	549 (79.5)	–	678	309 (87.1)	–

^a^similarity of nucleotide (nt) compared to rotavirus A strain 02V0002G3, rotavirus D strain 05 V0049 and rotavirus F strain 03 V0568

#### Caliciviridae

Two nearly complete chicken calicivirus genome sequences with lengths of 7865 and 7315 nt were found in samples 13D62 and 13Q45. Both contigs contained two coding open reading frames (polyprotein and the VP2 protein) and were closely related in a cluster of the genus Nacovirus (Fig. 2d).

#### Picobirnaviridae

Five picobirnavirus contigs were identified from samples 13D62 and 13Q45. Two or three distinct partial RNA-dependent RNA polymerase gene sequences were found in each sample. Three sequences were clustered with genogroup I, and the other sequence clustered with genogroup II (Fig. 2e).

### Detection of enteric viruses in the non-RSS-affected flock using multiplex RT-PCR and PCR

Intestinal samples from 86 non-RSS-affected broiler flocks were examined to detect enteric viruses using the developed multiplex RT-PCR method and PCR. Parvoviruses and chicken astroviruses were detected with very high positive detection rates of 75.6 and 62.8%, respectively (Table 4). Picornaviruses, caliciviruses and rotavirus species were also found to have low to moderate positive detection rates (7.0–39.5%).

**Table 4:** PCR experiment for detecting enteric viruses to non-viral enteritis broiler flock

Enteric virues	Number of positive flock / number of flock tested	Positive rate (%)
parvovirus	65/86	75.6
astrovirus	54/86	62.8
picornavirus	34/86	39.5
calicivirus	31/86	36.1
rotavirus A	25/86	29.1
rotavirus F	17/86	19.8
rotavirus D	6/86	7.0

## Discussion

High-throughput sequencing data generated by Illumina sequencing indicated the presence of several different viruses in four chicken flocks affected by RSS. Chicken astrovirus, parvovirus, calicivirus and rotavirus have been identified as causes of gastrointestinal tract infections in poultry, such as RSS, which is also called malabsorption syndrome [27]. A few studies using viral metagenomics could not specify any particular pathogen from the viral community in the chicken flocks with RSS because the distributions of enteric viruses in diseased and healthy flocks were not significantly different [17, 28, 29]. Chicken astrovirus has been distributed worldwide for decades and is nearly ubiquitous constituent of the chicken gut [30]. We also found a high positive detection rate (62.8%) for this virus in the non-RSS-affected chickens. On the other hand, metagenomics analysis in this study showed that only chicken astroviruses were common to chickens that had been diagnosed with RSS via pathological lesions in the crypts of the small intestine. In addition, chicken astrovirus was not identified in the fecal virome of healthy chickens in Brazil [31]. Chicken astrovirus has been suggested as an etiological agent for RSS, but it could be detected in chickens without microscopic lesions. Recently, the pathogenesis of chicken astrovirus in broilers was revealed. Serial passages of the virus from chicken to chicken induced the increased virulence, as shown by decreased weight gain and the presence of histopathological lesions [32]. Kang et al. provided strong evidence of chicken astrovirus as an etiological agent of RSS, although the mechanism for its reproduction via bird-to-bird passage is not known. Our metagenomics results support the notion of chicken astrovirus as a major etiology of RSS.

Chicken megriviruses of the *Picornaviridae* family detected in two samples tested in this study were shown to be similar to viruses identified from chickens with transmissible viral proventriculitis [33]. These cases were thought to be cases of RSS associated with proventriculitis [34], but the two cases did not exhibit histopathological lesions in the proventriculus. Viral sequences closely related to members of Gallivirus were identified, suggesting the diversity of chicken picornaviruses, but the role of these viruses in chicken disease is still unknown [35].

Parvovirus, calicivirus and rotavirus have been suggested as etiological agents of RSS in chickens [6, 7, 36] and were shown to be widely distributed in the non-RSS-affected chicken flocks in this study. As of yet, these viruses have not specified their pathogenesis and reproduced of the pathogenicity; therefore, further research is needed. Independently, we also identified the full NS gene sequence from chapparvovirus in a single chicken intestine sample. Chapparvovirus, which was recently classified, has been found in various vertebrate animals, such as birds, chickens, pigs, bats and dogs, throughout the world, and many studies have been performed to reveal the properties of this virus [37]. Although trials to identify the whole genome of this virus failed, we have the opportunity for novel viral discovery through environmental metagenomics.

In this study, chicken picobirnaviruses from the Republic of Korea were firstly identified using metagenomics, and viral sequences were grouped into genogroups I and II. Avian picobirnaviruses in genogroup I have primarily been noted to date, and those in genogroup II are rarely detected in chickens [17, 29, 38]. We could not design a primer set to detect picobirnaviruses due to the high genetic diversity between chicken picobirnaviruses. The high level of picobirnavirus sequence diversity in various hosts and environmental samples suggests the evolution of heterologous strains [39]. Coronavirus was found in sample 05D72, which was taken from chickens diagnosed with IB and RSS, and adenovirus was also found in sample 13D62, which was taken from chickens diagnosed with inclusion body hepatitis and RSS. Although the viral reads were not high in number, these findings indicate that viral metagenomics can help to determine the clinical etiological agents of chicken diseases.

## Conclusions

Chickens are a major protein source for human consumption, and their diseases are closely connected with public health concerns and economic losses. RSS in chickens is disease that impairs productivity in the Republic of Korea. In the present study, we used an unbiased metagenomic approach for viral pathogen discovery, the detection of novel viruses and the development of a molecular diagnostic tool to detect pathogens from the obtained sequences. We suggest that RSS in chickens, which is also called malabsorption syndrome, is caused by a chicken astrovirus. In addition, the multifactorial etiologies as a cause of RSS, as well as astrovirus enteritis could be ruled out through further studies of other enteric viruses, such as parvovirus, rotavirus and calicivirus.

## Supplementary information

**Additional file 1.** Base quality and filter result of high-throughput data obtained from samples in this study.

**Additional file 2.** Taxonomy annotation of viral reads obtained from samples in this study.

## Data Availability

The data generated or analyzed during this study are available from the corresponding author on reasonable request.

## Abbreviations

NS: Non structural polyprotein
nt: Nucleotide
PBS: Phosphate-buffered saline
PCR: Polymerase chain reaction
RACE: Rapid amplification of cDNA ends
RSS: Runting-stunting syndrome
RT: Reverse transcription
SPF: Specific pathogen free

## References

[1] Barners HJ, Guy JS (2003). Poultry enteritis-mortality syndrome: Iowa state press: Ames.

[2] Saif YM, Swayne DE, Glisson JR, LR MD, Nolan LK, Suarez DL, Nair V (2013). Viral enteric infections. Disease of Poultry.

[3] Pantin-Jackwood MJ, Swayne DE, Glisson JR, LR MD, Nolan LK, Suarez DL, Nair V (2013). Multicausal enteric disesase. Disease of Poultry.

[4] Randall CP, Reece RL, Mosby-Wolfe (1996). Color Atlas of Avian Histopathology.

[5] Songserm TH, Pol JMA, van Roozelaar D, Kok GL, Wagenaar F, ter Huurne AAHM (2003). A comparative study of the pathogenesis of malabsorption syndrome in broilers. Avian Dis.

[6] Otto P, Liebler-Tenorio EM, Elschner M, Reetz J, Lőhren U, Diller R (2006). Detection of rotaviruses and intestinal lesions in broiler chicks from flocks with runting and stunting syndrome (RSS). Avian Dis.

[7] Kisary J, Nagy B, Bitay Z (1984). Presence of parvoviruses in the intestine of chickens showing stunting syndrome. Avi Pathol.

[8] Baxendale W, Mebatsion T (2004). The isolation and characterization of astroviruses from chickens. Avi Pathol.

[9] Mettifogo E, Nuňez LFN, Chacón JL, Parra SHS, Astolfi-Ferreira CS, Jerez JA, Jones RC, Ferreira AJP (2014). Emergence of enteric viruses in production chickens is a concern for avian health. Sci World J.

[10] De Wit JJ, Dam GB, De Laar JM, Biermann Y, Verstegen I, Edens F, Schrier CC (2011). Detection and characterization of a new astrovirus in chicken and turkeys with enteric and locomotion disorders. Avian Pathol.

[11] Kouwenhoven B, Vertommen M, Goren E (1988). Investigations into the role of reovirus in the malabsorption syndrome. Avian Pathol.

[12] McNulty MS, Allan GM, McCracken RM (1983). Experimental infection of chickens with rotaviruses: clinical and virological findings. Avian Pathol.

[13] Meulemans G, Peeters JE, Halen P (1985). Experimental infection of broiler chickens with rotavirus. Br Vet J.

[14] Songserm T, Zekarias B, Van Roozelaar DJ, Kok RS, Pol JM, Pijpers AA, Ter Huurne AA (2002). Experimental reproduction of malabsorption syndrome with different combinations of reovirus, Escherichia coli, and treated homogenates obtained from broilers. Avian Dis.

[15] Ter Veen C, de Bruijn ND, Dijkman R, de Wit JJ (2017). Prevalence of histopathological intestinal lesions and enteric pathogens in Dutch commercial broilers with time. Avian Pathol.

[16] Van Loon AA, Koopman HC, Kosman W, Mumczur J, Szeleszczuk O, Karpinska E, Kosowska G, Lutticken D (2001). Isolation of a new serotype of avian reovirus associated with malabsorption syndrome in chickens. Vet Q.

[17] Day JM, Oakley BB, Seal BS, Zsak L (2015). Comparative analysis of the intestinal bacterial and RNA viral communities form sentinel birds placed on selected broiler chicken farms. PLoS One.

[18] Greninger AL (2018). A decade of RNA virus metagenomics is (not) enough. Virus Res.

[19] Edwards RA, Rohwer F (2005). Viral metagenomics. Nat Rev Microbiol.

[20] Stang A, Korn K, Wildner O, Űberla K (2005). Characterization of virus isolates by particle-asssociated nucleic acid PCR. J Clin Micobiol.

[21] Tamura K, Dudley J, Nei M, Kumar S (2007). MEGA4: molecular evolutionary genetics analysis (MEGA) software version 4.0. Mol Biol Evol.

[22] Maes P, Matthihnssens J, Rahman M, Van Ranst M (2009). RotaC: a web-based tool for the complete genome classification of group a rotaviruses. BMC Microbiol.

[23] Lűsebrink J, Schildgen V, Tillmann RL, Wittleben F, Bőhmer A, Műller A, Schildgen O (2011). Detection of head to-tail DNA sequences of human bocavirus in clinical samples. PLoS One.

[24] Bezerra DA, da Silva RR, Kaiano JH, Silvestre RV, de Souza OD, Linhares AC, Gabbay YB, Mascarenha JD (2012). Detection of avian group D rotavirus using the polymerase chain reaction for the VP6 gene. J Virol Methods.

[25] Zhao W, Hua XG, Yuan CL, Cui L, Shan TL, Dai XQ, Zhu AL, Yu Y, Zhu CX, Yang ZB (2011). Sequence analyses of the representative chinese-prevalent strain of avian nephritis virus in healthy chicken flocks. Avian Dis.

[26] Palinski RM, Mitra N, Hause BM (2016). Discovery of a novel parvovirinae virus, porcine parvovirus 7, by metagenomic sequecing of porcine rectal swabs. Virus Genes.

[27] Guy JS (1998). Virus infections of the gastrointestinal tract of poultry. Poulry Sci.

[28] Devaney R (2016). Trudgett Jm Trudgett a, Meharg C and Smyth V. a metagenomic comparison of endemic viruses from broiler chickens with runting-stunting syndrome and from normal birds. Avi Pathol.

[29] Lima DA, Cibulski SP, Tochetto C, Varela APM, Finkler F, Teixeira TF, Loiko MR, Cerva C, Junqueira DM, Mayer FQ, Roehe PM (2019). The intestinal virome of malabsorption syndrome-affected and unaffected broilers through shotgun metagenomics. Virus Res.

[30] Jackwood PMJ, Spackman E, Day JM, Rives D (2007). Periodic monitoring of commercial turkeys for enteric viruses indicates continuous presence of astrovirus and rotavirus on the farms. Avian Dis.

[31] Lima DA, Cibulski SP, Finkler F, Teixeira TF, Varela APM, Cerva C, Loiko MR, Scheffer CM, dos Santos HF, Mayer FQ, Roehe PM (2017). Faecal virome of healthy chickens reveals a large diversity of the eukaryote viral community, including novel circular ssDNA viruses. J Gen Virol.

[32] Kang KI (2018). Linnemann Em Icard Ahm Duraira V, Mund E, and sellers HS. Chicken astrovirus as an aetiological agent of runting-stunting syndrome in broiler chickens. J Gen Virol.

[33] Kim HR, Yoon SJ, Lee HS, Kwon YK (2015). Identification of a picornavirus from chickens with transmissible viral proventriculitis using metagenomic analysis. Arch Virol.

[34] Noiva R, Guy JS, Hauck R, Shivaprasad HL (2015). Runting stunting syndrome associated with transmissible viral proventriculitis in broiler chickens. Avian Dis.

[35] Boros A, Pankovics P, Adonyi A, Fenyvesi H, Day JM, Phan TG, Delwart E, Reuter G (2016). A diarrheic chicken simultaneously co-infected with multiple picornaviruses: complete genome analysis of avian picornaviruses representing up to six genera. Virology..

[36] Cubitt WD, Barrett ADT (1985). Propagation and preliminary characterization of a chicken candidate calicivirus. J Gen Virol.

[37] Jolly CJ, Lee Q, Padul MP, Pinello N, Williams SH, O’Rourke MB, Fumagalli MJ, Orkin JD, Shaban B, Brenner O, Weninger W, de Souza WM, Melin AD, Wong JJ, Crim MH, Monette S, and Roediger B. MKPV(aka MuCPV) and related chapparvoviruses are nephro-tropic and encode novel accessory proteins p15 and NS2. bioRxiv. 2019;732537; dio:10.1101/732537.

[38] Silva RR, Bezerra DAM, Kaiano JHL (2014). de Souza Oliveira, Silvestre RVD, Gabbay YB, Ganesh B, and Mascarenha JDP. Genogroup I avian picobirnavirus detected in Brazilian broiler chickens: a molecular epidemiology study. J Gen Virol.

[39] Ganesh B, Banyai K, Martella V, Jakab F, Masachessi G, Kobayashi N (2012). Picobirnavirus infections:viral persistence and zoonotic potential. Rev Med Viral.

