# Supplementary material for: Viral metagenomic analysis of chickens with runting-stunting syndrome in the Republic of Korea
Source: Virol J. 2020 Apr 15;17:53. doi: 10.1186/s12985-020-01307-z (PMC7157833; doi:10.1186/s12985-020-01307-z)
Supplement: Supplementary file 1 — Additional file 1. Base quality and filter result of high-throughput data obtained from samples in this study. [file 12985_2020_1307_MOESM1_ESM.docx]

Supplementary data 1. Base quality and filter result of high-throughput data obtained from samples in this study

| ID | Total Reads | Clean Reads | Filtered Reads | Clean Read Rate |
| --- | --- | --- | --- | --- |
| 05D72 | 19,381,670 | 14,893,900 | 4,487,770 | 76.85% |
| 07D11 | 21,387,788 | 17,152,624 | 4,235,164 | 80.20% |
| 13D62 | 24,907,152 | 19,711,846 | 5,195,306 | 79.14% |
| 13Q45 | 22,903,238 | 18,325,438 | 4,577,800 | 80.01% |
| Control | 22,813,882 | 18,906,096 | 3,907,786 | 82.87% |
