# Supplementary material for: Viral metagenomic analysis of chickens with runting-stunting syndrome in the Republic of Korea
Source: Virol J. 2020 Apr 15;17:53. doi: 10.1186/s12985-020-01307-z (PMC7157833; doi:10.1186/s12985-020-01307-z)
Supplement: Supplementary file 2 — Additional file 2. Taxonomy annotation of viral reads obtained from samples in this study. [file 12985_2020_1307_MOESM2_ESM.docx]

|  |  | **05D72** | **07D11** | **13D62** | **13Q45** | **control** |
| --- | --- | --- | --- | --- | --- | --- |
| **bacteriophage** | Leviviridae | 7,462 | 1,937 | 17,237 | 3,836 | 325,891 |
|  | MS2 | 7,060 | 1,937 | 13,921 | 3,836 | 270,026 |
|  | F1 | 403 | - | 2,960 | - | - |
|  | BZ13 | - | - | 43 | - | - |
|  | unassigned levivirus | - | - | 313 | - | 55,865 |
|  | Caudovirales | - | - | 89,149 | 6,402 | 9,235 |
|  | Siphoviridae | - | - | 82,207 | 3,248 | 71 |
|  | Myoviridae | - | - | 6,035 | 36 | 3,731 |
|  | Podoviridae | - | - | 907 | 3,118 | 5,433 |
|  | unassigned phage | - | 684 | 3,286 | 99 | 3,898 |
|  | **subtotal reads** | **7,463** | **2,618** | **109,672** | **10,337** | **339,024** |
| *Astroviridae* | avastrovirus | 13 | 1,097 | 736,160 | 2,115 | - |
|  | avian nephritis virus | 29 | 707 | 180,201 | 191 | - |
|  | chicken astrovirus | 86 | 1,278 | 264,458 | 1,902 | - |
|  | unassigned avastrovirus | - | 342 | 146,798 | - | - |
|  | feral pigeon astrovirus | - | - | 931 | - | - |
|  | **subtotal reads** | **128** | **3,424** | **1,328,548** | **4,208** | - |
| *Picornaviridae* | melegrivirus A | - | 19,399 | 135,369 | - | - |
|  | quail picornavirus | - | 6,119 | 35,167 | 2,782 | - |
|  | turkey hepatitis virus | - | 3,765 | 27,636 | - | - |
|  | pigeon picornavirus | - | - | 3,305 | 188 | - |
|  | salivirus sewage | - | - | - | 994 | - |
|  | turdivirus | - | - | 914 | 2,682- | - |
|  | gallivirus | 190,833 | 7,059 | 129 | 4,289 | - |
|  | enterovirus A | - | - | - | 17 | - |
|  | turkey gallivirus | 16,927 | 3,304 | - | 5,797 | - |
|  | equine rhinitis A | - | - | 3,292 | - | - |
|  | unassigned picornavirus | 6,888 | - | 1,688 | 1,818 | - |
|  | **subtotal reads** | **214,648** | **39,646** | **207,500** | **18,567** | - |
| *Parvoviridae* | chicken parvovirus | - | 807 | 5,817 | - | - |
|  | turkey parvovirus | - | 6,442 | 3,861 | 28 | - |
|  | adeno-associated virus | - | - | 9 | - | - |
|  | partridge parvovirus | - | - | - | 2,571 | - |
|  | unassainged parvovirus | - | 2,314 | 4,106 | - | - |
|  | **subtotal reads** | - | 9,563 | 13,793 | 2,599 | - |
| *Reoviridae* | rotavirus A | - | 2,647 | 40,757 | - | - |
|  | rotavirusF | - | 837 | 15,791 | - | - |
|  | rotavirusD | - | 104,182 | 10,723 | - | - |
|  | avian orthoreovirus | - |  | 187 | - | - |
|  | **subtotal reads** | - | **107,666** | **67,458** | - | - |
| *Caliciviridae* | chicken calicivirus | - | - | 18,592 | 7,822 | - |
|  | sapovirus | - | - | 684 | - | - |
|  | unassigned calicivirus | - | - | 526 | - | - |
|  | **subtotal reads** | - | - | 19,802 | 7,822 |  |
| *Picobirnaviridae* | human picobirnavirus | - | - | 7,882 | 5,397 | - |
|  | porcine picobirnavirus | - | - | 2,990 | - | - |
|  | fox picobirnavirus | - | - | 2,611 | - | - |
|  | otarine picobirnavirus | - | - | 1,694 | 49 | - |
|  | uncultured picobirnavirus | - | - | 424 | - | - |
|  | geoffroys-cat picobirnavirus | - | - | 33 | - | - |
|  | unassigned picorbirnavirus | - | - | 494 | - | - |
|  | **subtotal reads** | - | - | 16,128 | 5,446 | - |
| *Adenoviridae* | | - | - | 3,741 | - | - |
| *Coronaviridae* | | 409 | - | - | - | - |
| *Anelloviridae* | | 4,222 | - | - | - | - |
| **Total viral reads** | | **226,870** | **162,917** | **1,766,642** | **48,979** | **339,024** |

Supplementary data 2. Taxonomy annotation of viral reads obtained from samples in this study
